# Introductory

**Published:** 1845-06-01

**Authors:** 


### INTRODUCTORY.

On the occasion of introducing to the Medical Public the first number of a new Journal, it will be expected of the Editor to give some reasons for its appearance, and to premise some account of the objects to which it will be devoted.

With regard to the first, we have only to offer the conclusion, which has been formed, after due reflection, that a Medical Journal, if it commend itself to the approval, and interest of those to whom it will address itself for encouragement and support, may be satisfactorily sustained, and in various points of view, become subservient to useful ends. We might be somewhat distrustful of this conclusion, if it were peculiar to ourselves; but it is also entertained by several professional brethren, who have felt sufficient confidence in its correctness, to unite in originating this undertaking, and pledge themselves to secure a fair trial of its practicability.

It will readily be acknowledged, that for the more voluminous and elaborate Journals, in medical as in other departments of knowledge, we must look to the larger cities, where the elements and facilities for their preparation and diffusion, are to be found in the greatest abundance. But without any derogation from the superior claims of these, there are many reasons why they do not, and cannot accomplish all the objects to be derived from periodical literature. Of these reasons, we may hereafter, take occasion to speak more particularly, and to discuss their respective merits. But assuming, for the present, their existence, and validity, it has seemed to ourselves and others, that Buffalo, is in many respects, a desirable location for a Medical Journal. This opinion is based on its present and prospective size and resources; its relations with the east and west, through canals and rail-roads on the one hand, and the chain of the great Lakes, with their numerous tributaries, on the other. It is believed that sufficient material to commence an enterprise of this kind, may be derived from sources which will be constantly increasing and improving; and that a Medical Journal may do much, not only toward making available the material which already exists, but to render its future availibility and improvement commensurate with its increase. By means of facilities for rapid and extensive communications on every side, our location is peculiarly favorable for the collection and diffusion of facts from a wide circuit, and the interchange of views and opinions among members of the profession, not only in this section of country, but situated at points remotely distant from each other.

We have also, in this project, ventured to act upon the belief, (and we hope it may be expressed without undue presumption,) that the Medical Fraternity, whose interest and assistance will be especially solicited, are not deficient in the ability, or the disposition to contribute their proportion of effort for the advancement of the legitimate ends and interests of our common Science.

With these views, the Journal is commenced. As before intimated, its continuance is secured for a sufficient period, to provide a practical test of its feasibility and utility. The result of the experiment will determine whether it is to be abandoned as a premature or misplaced project ; or on the other hand, whether it is to be continued on a larger scale.

In conducting the Journal, we shall in the first place, devote as much space as practicable to original communications. Cases are constantly occuring under the observation of practical men, which are interesting from their novelty, and which contribute either to develop or confirm important truth. We shall be glad to receive reports of such cases, and communicate them for the benefit of the whole profession. A great number of useful thoughts and ideas, originate in the minds of observing and reflecting Practitioners, which it is desirable to throw into the crucible of Medical literature, there to produce results according to their intrinsic value. We offer our Journal as a medium of communication, in this respect between the minds of individuals, and the common mind of the profession. Of particular points of Medical science, upon which we may especially desire to collect information, we shall take occasion to speak from time to time, as they may suggest themselves : But in general terms, the first object will be to gather up from different sources important matter, much of which would otherwise be lost, adding it to the common stock of knowledge, and, thereby contributing to the progress of Medical Science.

In the second place, we shall endeavour to supply to those who may become our Patrons, condensed intelligence of discoveries, improvements and new views in Medicine, Surgery, and collateral branches of science, which may be communicated to the profession in this country or abroad, through periodicals and books. We shall have access to several of the most prominent Journals, Domestic and Foreign, and shall avail ourselves of their contents, for the benefit of our readers. In doing this, we shall not undertake to conflict with their individual claims to the esteem and patronage of the profession, still less, in any measure, to supersede the importance of resorting to them more directly as sources of valuable information. So far from this, we shall strive to promote their circulation, by endeavouring to diffuse a more general appreciation of their value.

Our pages will be open to interchange of opinions, and free discussion, not only of subjects strictly belonging to Medical Science—but of any involved in the progress, or interests of the Medical profession. Medical Education, Medical Legislation, Quackery, Medical Ethics, and the various plans of Medical reform, are among the Subjects, which at the present time, furnish questions of interest and moment, both as regards the profession and the Public. We shall be desirous of appropriating to articles upon these topics, as much space as our limits will allow, and to afford an opportunity for the expression of different views and opinions, without restraint, excepting by the rules of decorum and propriety.

We would add that the Journal is pledged to no interests apart from those which relate exclusively to the progress of Medical Science, and the advancement of the Medical profession. It is not instituted for any sectional objects, or partizan views; but to serve as an organ for the impartial and untrammelled utterance of opinion on any matters pertaining directly or indirectly to its professed objects.

In conclusion, the Editor desires to say, in his own behalf, that he looks for the means of success in this undertaking, not to his own exertions, so much as to the assistance which he expects to receive from others; he enters upon the duty, however, with a determination to do what he can personally to contribute to its success, and is stimulated with the hope that this will be such as to authorize, ere long the enlargement of the Journal, so that he may be able to render it much more acceptable and useful than is compatible with its present restricted limits.

*Buffalo, June 1st*, 1845.

